# Glioblastoma in older adults

**DOI:** 10.18632/aging.101377

**Published:** 2018-02-01

**Authors:** Matthias Holdhoff, Raphael Rothenberger, Ilene E. Browner

**Affiliations:** 1 Brain Cancer Program, Sidney Kimmel Comprehensive Cancer Center at Johns Hopkins, Johns Hopkins University School of Medicine, Baltimore, MD 21287, USA; 2 Department of Oncology, Sidney Kimmel Comprehensive Cancer Center at Johns Hopkins, Johns Hopkins University School of Medicine, Baltimore, MD 21287, USA

**Keywords:** glioblastoma, elderly, radiation, hypofractionated radiation, temozolomide

Glioblastoma (GBM) is the most common primary brain cancer in adults. Median age at diagnosis is about 60-62 years and approximately 45% of patients are over the age of 65, highlighting that this is a disease of older adults [1]. The standard of care for newly diagnosed GBM was defined by the European Organization for Research and Treatment of Cancer (EORTC)/National Cancer Institute of Canada (NCIC) study, published in 2005, that showed that chemoradiation with 60 Gy of radiation (RT) in 30 fractions plus temozolomide (TMZ) followed by 6 months of adjuvant TMZ improved survival compared with RT alone [2]. This study, however, excluded patients over the age of 70 due to concern about toxicity. Hence, questions about optimal treatment of older patients remained, leading to several randomized trials that focused on this patient population that have shed more light into the role of postoperative management of older patients with GBM. The first study to illustrate a benefit from post-operative treatment in this population compared RT alone with best supportive care [3]. Median overall survival was poor but better in the treatment arm: 6.7 versus 3.9 months, respectively. Thereafter, two European studies were published, comparing single modality therapy with TMZ, hypofractionated (hRT) or standard RT (RT) [4,5]. Compared to the original EORTC/NCIC study (in patients aged 18-70), survival was considerably short and there was no significant difference between the different monotherapies. None of these trials included a combination RT and TMZ arm and the question about a role for combination therapy in this setting remained unanswered. The studies however demonstrated favorable outcomes in patients with O^6^-methylguanine-DNA-methyltransferase (MGMT*)* promoter methylated GBM compared with those that were unmethylated. MGMT methylation is an established positive prognostic marker that also predicts for a higher likelihood of benefit from TMZ, and prior studies illustrated that the prevalence of this marker is similar in older compared to younger patients with GBM [1]. The question about combination therapy in older patients with GBM has been addressed by a recently published EORTC/NCIC study that was conducted in patients aged 65 and older using a similar design to the landmark EORTC/NCIC study [6]. There was, however, one main difference in the design: instead of the standard 6 weeks of chemoradiation, patients received a shorter course of radiation, hRT (40 Gy in 15 fractions), considering less short-term toxicity with this regimen as compared to the longer course of chemoradiation. Similarly to the original combination therapy study in patients aged 18-70, this trial showed a significant benefit from the addition of TMZ to RT. The use of hRT and not standard RT was based on a trial in older patients comparing hRT and RT as monotherapy, that showed similar survival [7]. Compared to the previous study however, overall survival was inferior with 9.3 months in the elderly GBM study versus 14.6 months in the original GBM protocol that excluded patients over the age of 70 [6,2]. On first look, this difference appears obvious as advanced age is a known adverse prognostic factor and older people have more co-morbidities and other competing risk factors. The relative benefit from TMZ however was similar in both trials. Of particular concern is that patients receiving short course, hRT only received 50% of the TMZ concomitantly with RT compared patients receiving the 6-week course. The impact of this is unknown. To date, the two regimens have not yet been prospectively compared in the older GBM population and it remains unclear which of the two regimens may be superior. Based on this, we argue that standard 6-week chemoradiation as defined by the original EORTC/NCIC study is a reasonable treatment approach for patients who are considered well enough to tolerate this regimen.

As for any older patient with cancer, treatment decisions need to be made on an individual basis, considering all aspects of the patient’s clinical status, including age, but also considering performance status, organ function and overall functional reserve.
